# The Role of Serum C-Reactive Protein Measured by High-Sensitive Method in Thyroid Disease

**DOI:** 10.1007/s00005-014-0282-1

**Published:** 2014-05-04

**Authors:** Agata Czarnywojtek, Maciej Owecki, Małgorzata Zgorzalewicz-Stachowiak, Kosma Woliński, Ewelina Szczepanek-Parulska, Bartłomiej Budny, Ewa Florek, Joanna Waligórska-Stachura, Izabela Miechowicz, Maciej Bączyk, Nadia Sawicka, Sumit Dhir, Marek Ruchała

**Affiliations:** 1 Department of Endocrinology, Metabolism and Internal Medicine, University of Medical Sciences, Przybyszewskiego 49, 60-355 Poznan, Poland; 2 Laboratory of Medical Electrodiagnostics, Department of Health Prophylaxis, University of Medical Sciences, Poznan, Poland; 3 Laboratory of Environmental Research, Department of Toxicology, University of Medical Sciences, Poznan, Poland; 4 Department of Computer Science and Statistics, University of Medical Sciences, Poznan, Poland; 5 University of Medical Sciences, Poznan, Poland

**Keywords:** Hs-CRP, Thyroid disease, Hashimoto’s thyroiditis, Graves’ disease, Thyroid cancer

## Abstract

The aim of this study was the evaluation of serum C-reactive protein (CRP) concentration as a marker of the inflammatory state in many different thyroid diseases and its dependence on the stage and duration of disease. We conducted a retrospective analysis of 444 randomly selected patients with different kinds of thyroid disease (106 men and 338 women, ranging 18–72 years of age; mean 56.2 ± 5.0 years; median 52 years). Group 1 (G1) comprised 250 patients with hyperthyroidism. Group 2 (G2) consisted of 72 euthyroid patients. Group 3 (G3) consisted of 122 patients with hypothyroidism. Free T4, free T3, and thyrotropin (TSH) levels were measured using the electrochemiluminescent method. Human serum thyroglobulin autoantibodies (Tg-Abs), thyroperoxidase autoantibodies (TPO-Abs), and autoantibodies against the thyrotropin receptor (TSHR-Abs) levels were measured by radioimmunoassay. The high-sensitive CRP (Hs-CRP) level (reference range <3 mg/L) was determined with a highly sensitive latex-based immunoassay. The mean value of Hs-CRP in G1 was 3.6 ± 2.8 mg/L, in G2 2.5 ± 1.5 mg/L and in G3 5.9 ± 5.8 mg/L. Hs-CRP (in mg/L) medians, interquartile and the total ranges in G1 were 3.0 (2.0 [0.1–21.0] 4.0); in G2: 2.3 [1.8 (0.2–9.2) 3.2]; and in G3: 4.3 [2.2 (0.3–31.5) 7.8]. We found statistically significant differences (Kruskal–Wallis test) in serum Hs-CRP values between G1 and G2 (*P* = 0.007), G1 and G3 (*P* = 0.001), G2 and G3 (*P* < 0.001). In G1, statistically significant correlation was confirmed between Hs-CRP and Tg-Abs (*r* = −0.22, *P* = 0.0016), CRP and TPO-Abs (*r* = −0.26, *P* < 0.001), and also between Hs-CRP and TSHR-Abs (*r* = −0.18, *P* = 0.02). In the remaining cases, differences between Hs-CRP and TSH levels (*r* = −0.09, *P* = 0.16) were not statistically significant. In G2, no statistically significant correlation was observed: Hs-CRP and Tg-Abs (*r* = −0.18, *P* = 0.13), Hs-CRP and TPO-Abs (*r* = −0.17, *P* = 0.15), Hs-CRP and TSH (*r* = 0.01, *P* = 0.91), Hs-CRP and TSHR-Abs (*r* = −0.19, *P* = 0.17). In G3, a statistically significant correlation was confirmed between Hs-CRP and Tg-Abs (*r* = 0.22, *P* = 0.012), Hs-CRP and TSH (*r* = −0.28, *P* = 0.001). No statistically significant correlation was observed between Hs-CRP and TPO-Abs (*r* = 0.20, *P* = 0.06) and between Hs-CRP and TSHR-Abs (*r* = −0.23, *P* = 0.11). Hs-CRP is increased in various types of hypothyroidism. This is particularly relevant in postpartum thyroiditis and in patients after radioiodine treatment. The impact of this situation on human health requires further research, however, one might assume that some types of thyroid disease may lead to systemic inflammatory reactions that are reflected in elevated CRP levels.

## Introduction

C-reactive protein (CRP) is an acute-phase plasma protein composed of five identical polypeptide subunits of 206 amino acids each, discovered by Tillett and Francis (1930). The CRP concentration in plasma rises due to a response to cytokines in the plasma (IL-1β and IL-6) that are produced predominantly by macrophages (Lau et al. 2005), as well as adipocytes. CRP binds to phosphocholine on microbes playing a part in complement binding and phagocytosis by macrophages (opsonin-mediated phagocytosis) of foreign and damaged cells, and also plays an important role in the early immune defense against infections through innate immunity. CRP may increase to over 50,000 times the normal value, starting within 6 h and reaching a peak in two days depending on the degree of tissue damage and inflammation. Its concentration depends on the rate of synthesis, since it has a constant half-life in plasma (Pepys 1981; Pepys and Hirschfield 2003).

As a sensitive marker of inflammation, it is associated with an increased rate of cardiovascular disease and mortality (Koenig et al. 2008; Strandberg and Tilvis 2000). Several reports have identified the assessment of plasma CRP as a predictor of metabolic syndrome and type 2 diabetes mellitus (Han et al. 2002; Laaksonen et al. 2004; Onat et al. 2008; Pradhan et al. 2001). The inhibition of CRP has been considered as a treatment for myocardial and cerebral infarcts due to the complementary role that CRP plays in enhancing ischemic necrosis, but has only been tested in animals to date (Ridker et al. 2008). Although the causal link between cancer and inflammation is not clear, chronic inflammation does increase cancer susceptibility in some organs. The role of anti-inflammatory medication, and thereby the reduction of CRP, in reducing cancer risk has been suggested in colon cancer, with a statistically significant difference in CRP seen between the absence and presence of colon cancer (Baron et al. 2003; Erlinger et al. 2004). It has also been suggested that the level of CRP may be used as a measure of arterial hardening and as an indicator to gauge its amelioration in hypothyroid patients (Nagasaki et al. 2007). However, CRP is not a routinely measured parameter in the diagnosis of thyroid diseases, even though due attention should be given to it in the presence of thyroiditis, whether in the course of Hashimoto’s disease, hyperthyroidism or hypothyroidism induced by interferon (IFN)-α or amiodarone (AM).

The aim of this study was to approach the evaluation of serum high-sensitive CRP (Hs-CRP) concentration as a marker of the inflammatory state in many different thyroid diseases and its dependence on the stage and duration of disease.

## Materials and Methods

### Patients

We conducted a retrospective analysis of 444 randomly selected patients with different kinds of thyroid disease (106 men and 338 women, ranging 18–72 years of age; mean 56.2 ± 5.0 years; median 52 years) who were treated or diagnosed in our Department, both in out- and in-patient settings (284 and 160 patients, respectively). Their common feature was that all of them suffered from thyroid disorders. However, our study recruited individuals with different kinds of thyroid disease, so that we were able to compare Hs-CRP between different subgroups. Consequently, the criteria of randomization were set as to achieve groups of patients suffering from various thyroid disorders in similar numbers, but even in spite of this, we did not obtain indifferent numbers of subjects in subgroups; those numbers ranged 10–57. This discrepancy was caused by the rare prevalence of some types of thyroid disorders. The exclusion criteria were cigarette smoking, injuries, cancer (other than thyroid cancer), rheumatic diseases, and tooth disease. Hs-CRP values for patients after menopause receiving hormonal replacement therapy were highlighted.

Group 1 (G1) comprised 250 patients with hyperthyroidism divided into several subgroups: A—Graves’ disease (GD; *n* = 24; 10.6 %); B—GD with thyroid-associated ophthalmopathy (*n* = 24; 10.6 %); C—toxic nodular goiter (TNG; *n* = 24; 9.2 %); D—toxic adenoma (*n* = 24; 10.6 %); E—IFN-α-induced thyrotoxicosis (IIT) (*n* = 25; 11.0 %); F—type I amiodarone-induced thyrotoxicosis (AIT) with high radioactive iodine uptake (RAIU; *n* = 57; 25.2 %); G—type II AIT with low RAIU (*n* = 36; 15.9 %); H—hyperthyroid phase in papillary carcinoma after radioiodine therapy (RIT) during suppression (*n* = 36; 15.9 %).

Group 2 (G2) comprised 72 euthyroid patients: I—GD during therapy (for at least 6 months) with thiamazole (*n* = 24; 33.3 %); J—Hashimoto’s thyroiditis (HT) during therapy with l-thyroxine (*n* = 24; 33.3 %); K—TNG treated for at least 3 months with RIT (*n* = 24; 33.4 %).

Group 3 (G3) comprised 122 patients with hypothyroidism: L—TNG after 1 year of RIT (*n* = 24; 19.2 %); M—HT (*n* = 24; 19.2 %); N—postpartum thyroiditis, 6 months after childbirth (*n* = 14; 13.6 %); O—IFN-α-induced hypothyroidism (IIH; *n* = 24; 19.2 %); P—hypothyroid phase in papillary carcinoma 1 month after total thyroidectomy (*n* = 36; 28.8 %).

Hyperthyroidism was diagnosed on the basis of elevated free thyroid hormones (TH), and suppressed thyrotropin (TSH). Subclinical hyperthyroidism was defined as normal free TH, and suppressed TSH. In addition, the following tests were used to classify patients into subgroups: elevated 24-h RAIU, a positive titer of circulating thyroid autoantibodies against thyroglobulin, thyroidal peroxidase, and TSH receptor (abbreviation, respectively, Tg-Abs, TPO-Abs, TSHR-Abs), or a negative titer of circulating thyroid autoantibodies (seen in TNG, toxic adenoma, AIT type I, AIT mixed), and decreased 24-h RAIU (seen in AIT type II, IIH). The Graves’ ophthalmopathy was diagnosed according to the EUGOGO consensus (Bartalena et al. 2008) in Polish modification (Bednarczuk et al. 2009).

The diagnosis of hypothyroidism was based on the following criteria: decreased free TH, and elevated TSH. Similarly to the hyperthyroid group, the following tests were used to classify patients into subgroups: RAIU, TPO-Abs and Tg-Abs, and decreased volume of the thyroid without relevant nodules (≥1 cm) on conventional ultrasonography.

### Experimental Protocol

Most of the participants (hyperthyroid patients) attended our Department for the purpose of being adequately prepared for RIT; the rest of the thyroid patients were recruited from an out-patient clinic. For estimation of the inflammatory process, we measured Hs-CRP in blood. The study protocol was approved by the local ethics committee.

### Laboratory Methods

Free T4, free T3, as well as TSH, were measured by the electrochemiluminescent method (Roche, Switzerland). Serum TSH concentration was measured with a third-generation sensitivity ≤0.005 IU/mL. Serum Tg-Abs, TPO-Abs and TSHR-Abs were measured by radioimmunoassay (Brahms, Germany). Standard laboratory values in our laboratories were as follows: free T4 11.5–21.5 pmol/L, free T3 3.9–6.8 pmol/L, TSH 0.27–4.2 μIU/mL, TSHR-Abs <2 IU/L, TPO-Abs 0–34 IU/mL and Tg-Abs 10–115 IU/mL. The Hs-CRP level (reference range <3 mg/L) was determined with a highly sensitive latex-based immunoassay (Dade Behring, Newark, DE, USA; sensitivity 0.05 mg/L) (Whicher et al. 1994).

### Treatment

Beta-blockers were used as first-line therapy for symptomatic treatment (G1: A, B, D, H, E) of patients with destructive thyrotoxicosis and GD when tachycardia occurred as an adverse reaction to antithyroid drug (ATD) therapy. ATDs, methimazole or propylthiouracil were applied for at least 6 months to achieve remission (G2: I). Radioiodine (RAI) was used after 1 year of ineffective therapy, when ATD therapy was contraindicated (agranulocytosis, thrombocytopenia, transaminasemia) and also in the case of thyroid cancer (G1: H). Substitution therapy with l-thyroxine was administered to compensate for hypothyroidism in HT (subgroup: O; G3) or after RIT (G3: P or subgroup P; G3 (use one)). The patients with IIT received IFN-α-2b (Roferon-A, Hoffman-LaRoche Inc., Basel, Switzerland) or pegylated-IFN-α-2b in a combination with ribavirin (Rebetol; Schering-Plough, Germany) (G1: E), a similar combination which was used in the cases of AM (G1: F, G).

### Statistical Analysis

The calculations were performed using Statistica 10 from StatSoft. The *P* level of <0.05 was considered statistically significant. The Kruskal–Wallis test was used because of the lack of normal distribution of Hs-CRP values in individual groups. Studying the influence of Hs-CRP on the parameters such as autoantibodies (Tg-Abs, TPO-Abs, TSHR-Abs) and TSH, a Pearson linear correlation was used when variable distributions were normal, and Spearman’s rank correlation when variables were not normally distributed.

## Results

### Serum TH, TSH and Autoantibodies Level

The general characteristics of the participants (106 men and 338 women) divided into groups G1, G2, G3 are summarized in Table 1. The results with regard to a more detailed division into subgroups termed A–P are shown in Table 2 and can be summarized as follows: the highest concentrations of free tetraiodothyronine (fT4; 31.4 ± 6.9 pmol/L) and free triiodothyronine (fT3; 13.6 ± 4.3 pmol/L) were found in hyperthyroid patients (G1) with type I AIT (subgroup F) while the lowest fT4 (6.5 ± 2.7 pmol/L) and fT3 (4.1 ± 1.9 pmol/L) were observed in G3 (HT, subgroup M). The level of TSH was the lowest in subgroup F (TSH 0.005 μIU/mL) and extremely high in the case of subgroup M (HT, G3) (77.2 ± 31.3 μIU/mL). TPO-Abs, Tg-Abs, or both were positive for most cases in both G1 and G3. The highest concentration of TPO-Abs was found in subgroup M of G3 (TPO-Abs 882.8 ± 371.7 IU/mL). In subgroups A and B (G1), the mean Tg-Abs and TPO-Abs levels were not significantly different from the others. TSHR antibodies were detected only in the samples of subgroup B (G1). Similarly, there were no elevated levels of TSHR-Abs in G2 and G3.

**Table 1:** Clinical characteristics of studied group of patients with hyperthyroidism (G1), euthyroidism (G2) and hypothyroidism (G3)

	G1	G2	G3	G1 vs. G2 (*P* value)	G1 vs. G3 (*P* value)	G2 vs. G3 (*P* value)
Total no. of patients	250	72	122	NS	NS	NS
Number of M:F	51:199	21:51	34:88	NS	NS	NS
Age (year)	56.3 ± 13.7	47.6 ± 27.3	39.3 ± 21.1	NS	NS	NS
fT3 (pmol/L)	6.9 ± 3.6	5.3 ± 1.2	3.8 ± 1.7	NS	NS	NS
fT4 (pmol/L)	21.4 ± 9.6	14.3 ± 4.4	12.9 ± 4.0	NS	NS	NS
TSH (μIU/mL)	0.1 ± 0.1	5.5 ± 12.3	30.0 ± 36.4	NS	NS	NS
TPO-Abs (IU/mL)	639.1 ± 1,050.1	551.1 ± 586.9	509.8 ± 656.1	NS	<0.001	NS
Tg-Abs (IU/mL)	106.9 ± 262.8	174.9 ± 340.9	175.6 ± 320.6	NS	NS	NS
TSHR-Abs (U/L)	3.0 ± 6.4	1.4 ± 1.3	0.3 ± 0.2	<0.05	NS	NS
Hs-CRP (mg/L)	3.6 ± 2.8	2.5 ± 1.5	5.9 ± 5.8	0.007	0.001	<0.001

Data are given as mean ± SD. The reference ranges for serum hormone and autoantibody concentrations in our laboratory were as follows: TSH 0.27–4.2 μIU/mL, fT4 11.5–21.5 pmol/L, fT3 3.9–6.8 pmol/L, TSHR-Abs <2 IU/L, Tg-Abs 10–115 IU/mL, TPO-Abs 0–34 IU/mL, Hs-CRP <3 mg/L

*NS* not significant statistically

**Table 2:** Clinical characteristics of all studied subgroup (from A to P) of patients with hyperthyroidism (G1), euthyroidism (G2) and hypothyroidism (G3)

Subgroups	fT3 (pmol/L)	fT4 (pmol/L)	TSH (μIU/mL)	TPO-Abs (IU/mL)	Tg-Abs (IU/mL)	TRAK (IU/L)	Hs-CRP (mg/L)
G1							
A	7.9 ± 3.6	24.3 ± 4.7	0.0 ± 0.1	1,806.3 ± 1,100.4	379.8 ± 310.9	2.7 ± 0.7	4.0 ± 3.9
B	12.4 ± 9.6	23.3 ± 4.4	0.2 ± 0.0	1,524.6 ± 1,216.5	164.8 ± 306.8	15.0 ± 10.6	3.0 ± 1.1
C	7.5 ± 0.9	25.5 ± 3.3	0.0 ± 0.0	44.8 ± 7.7	39.5 ± 11.4	–	3.0 ± 4.1
D	11.4 ± 9.6	27.3 ± 4.4	0.1 ± 0.2	41.5 ± 16.7	27.3 ± 18.9	–	2.5 ± 1.5
E	10.1 ± 3.1	25.5 ± 3.9	0.1 ± 0.0	1,464.1 ± 1,228.7	159.4 ± 301.6	0.2 ± 0.2	2.9 ± 1.1
F	15.9 ± 4.7	32.9 ± 4.3	0.0 ± 0.0	42.0 ± 19.4	27.9 ± 5.7	0.7 ± 0.6	4.9 ± 3.3
G	13.0 ± 2.9	31.3 ± 2.4	0.0 ± 0.0	41.5 ± 20.9	28.0 ± 17.1	1.4 ± 1.3	5.5 ± 3.8
H	7.1 ± 3.6	23.3 ± 1.2	0.1 ± 0.1	–	–	–	2.3 ± 2.7
G2							
I	5.1 ± 2.6	14.3 ± 4.4	1.5 ± 0.8	882.8 ± 371.7	362.6 ± 481.8	2.6 ± 0.6	2.0 ± 1.2
J	4.3 ± 0.3	17.3 ± 5.1	1.3 ± 0.4	729.0 ± 700.1	134.6 ± 225.0	0.2 ± 0.1	2.7 ± 2.0
K	5.1 ± 2.2	16.3 ± 2.4	1.4 ± 0.9	27.3 ± 18.9	41.5 ± 16.7	0.3 ± 0.1	2.5 ± 1.1
G3							
L	5.7 ± 3.9	10.4 ± 1.4	13.5 ± 19.0	729.0 ± 711.3	134.6 ± 255.0	0.2 ± 0.2	10.8 ± 7.8
M	6.4 ± 2.2	8.3 ± 2.1	9.4 ± 8.8	1,070.5 ± 621.6	567.2 ± 363.6	–	8.6 ± 3.9
N	7.3 ± 1.4	10.1 ± 0.4	7.7 ± 6.2	42.8 ± 18.9	29.6 ± 23.8	–	6.8 ± 4.3
O	7.6 ± 1.9	8.4 ± 1.8	9.7 ± 7.2	430.4 ± 625.1	193.9 ± 263.6	0.3 ± 0.2	2.7 ± 0.9
P	9.3 ± 3.5	7.9 ± 3.7	77.3 ± 31.3	–	135.7 ± 372.3	–	2.9 ± 5.2

Data are given as mean ± SD. The reference ranges for serum hormone and autoantibody concentrations in our laboratory were as follows: TSH 0.27–4.2 μIU/mL, fT4 11.5–21.5 pmol/L, fT3 3.9–6.8 pmol/L, TSHR-Abs <2 IU/L, Tg-Abs 10–115 IU/mL, TPO-Abs 0–34 IU/mL, Hs-CRP <3 mg/L

*NS* not significant statistically, *A* Graves’disease (GD), *B* GD with thyroid-associated ophthalmopathy, *C* TNG, *D* toxic adenoma, *E* IFN-α-induced thyrotoxicosis, *F* type I AIT, *G* type II AIT with low RAIU, *H* hyperthyroid phase in papillary carcinoma after RIT during suppression, *I* GD during therapy (for at least 6 months) with thiamazole, *J* HT during therapy with l-thyroxine, *K* TNG treated for at least 3 months with RIT, *L* TNG after 1 year of RIT, *M* HT, *N* postpartum thyroiditis, 6 months after childbirth, *O* IFN-α-induced hypothyroidism, *P* hypothyroid phase in papillary carcinoma 1 month after total thyroidectomy

### Serum Hs-CRP Level in the Studied Group

The mean values of Hs-CRP were: in G1 3.6 ± 2.8 mg/L, in G2 2.5 ± 1.5 mg/L and in G3 5.9 ± 5.8 mg/L.

**Fig. 1:**
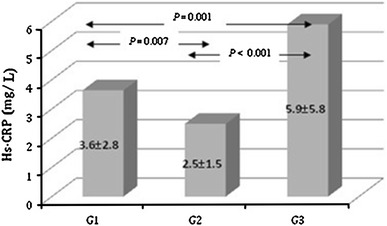
Mean concentrations and standard deviations of Hs-CRP serum levels in the studied group of patients with hyperthyroidism (G1), euthyroidism (G2) and hypothyroidism (G3). Hs-CRP <3 mg/L

Hs-CRP (in mg/L) medians, interquartile and total ranges in G1 were 3.0 [2.0 (0.1–21.0) 4.0]; in G2 they were 2.3 [1.8 (0.2–9.2) 3.2]; and in G3 they were 4.3 [2.2 (0.3–31.5) 7.8], respectively (Table 1). We found statistically significant differences (Kruskal–Wallis test) in serum Hs-CRP values between G1 and G2 (*P* = 0.007), G1 and G3 (*P* = 0.001), G2 and G3 (*P* < 0.001) as shown in Fig. 1. The results with regard to a more detailed division into subgroups termed A–P are shown in Table 2.

Kruskall–Wallis test revealed significant differences in CRP concentration between subgroups of G1 and G3 groups. Comparisons between these subgroups of G1 and G3 are shown in Tables 3 and 4. We have also found significant difference between two subgroups encompassing patients after RIT—K and L (*P* < 0.001, Mann–Whitney test).

**Table 3:** *P* values for comparison of subgroups of group G1 (Kruskall–Wallis test)

	A	B	C	D	E	F	G	H
A		0.62	0.79	1.00	0.76	0.00	0.00	1.00
B	0.62		0.23	1.00	1.00	0.29	0.23	0.33
C	0.79	0.23		1.00	0.05	0.09	0.13	0.00
D	1.00	1.00	1.00		1.00	0.00	0.00	1.00
E	0.00	1.00	0.05	1.00		0.18	0.15	0.41
F	0.00	0.29	0.09	0.00	0.18		1.00	0.00
G	0.00	0.23	0.13	0.00	0.15	1.00		0.00
H	1.00	0.33	0.00	1.00	0.41	0.00	0.00

**Table 4:** *P* values for comparison of subgroups of group G3 (Kruskall–Wallis test)

	L	M	N	O	P
L		1.00	0.74	0.00	0.00
M	1.00		1.00	0.00	0.00
N	0.74	1.00		0.00	0.00
O	0.00	0.00	0.00		1.00
P	0.00	0.00	0.00	1.00	

### Correlations between Serum Hs-CRP Levels and Various Parameters (TPO-Abs, Tg-Abs, TSHR-Abs) in the Studied Group

**Fig. 2:**
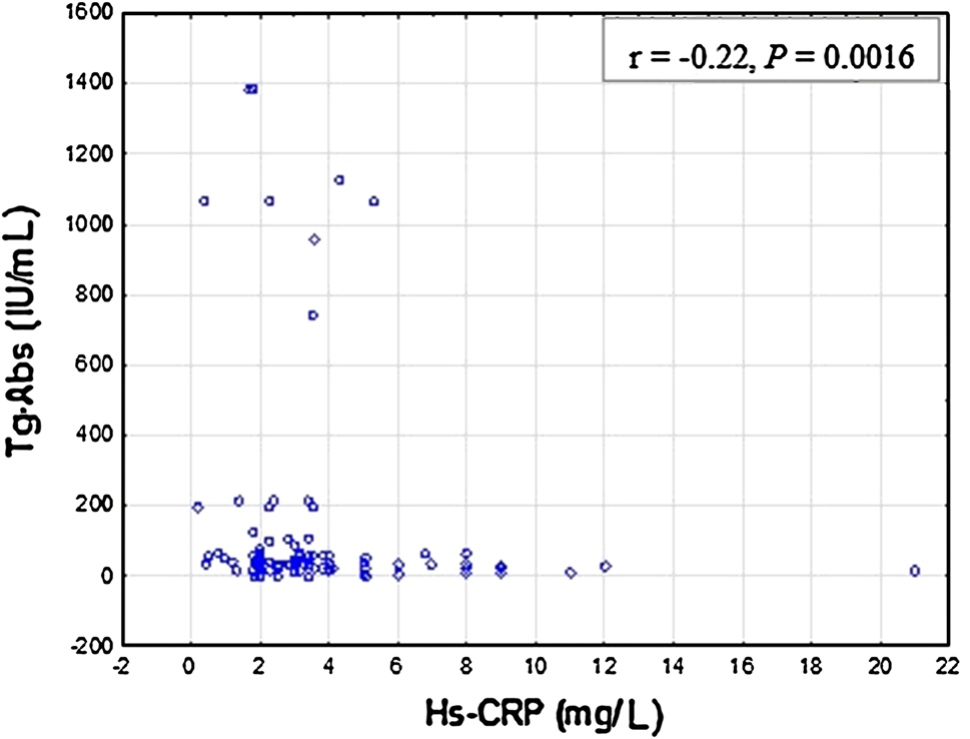
Correlations between serum Hs-CRP and Tg-Abs levels in hyperthyroid patients (G1). Data are given as mean ± SD. Tg-Abs 0–34 IU/mL; Hs-CRP <3 mg/L. Spearman’s rank correlation (*r* = −0.22, *P* = 0.0016) was employed to assess the relationship between mean Hs-CRP and Tg-Abs levels

**Fig. 3:**
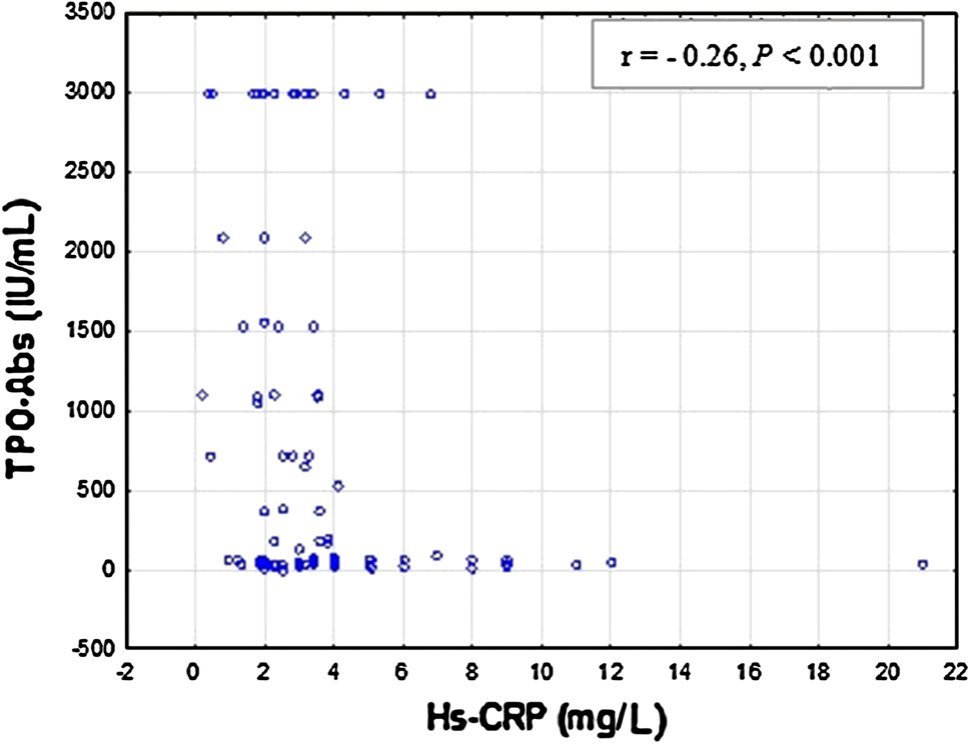
Correlations between serum Hs-CRP and TPO-Abs levels in hyperthyroid patients (G1). TPO-Abs 10–115 IU/mL; Hs-CRP <3 mg/L. Spearman’s rank correlation (*r* = −0.26, *P* < 0.001) was employed to assess the relationship between Hs-CRP and TPO-Abs levels

**Fig. 4:**
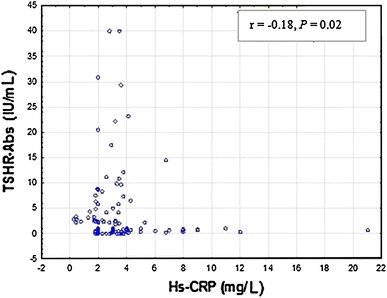
Correlations between serum Hs-CRP and TSHR-Abs levels in hyperthyroid patients (G1). TSHR-Abs <2 IU/L; Hs-CRP <3 mg/L. Spearman’s rank correlation *r* = −0.18, *P* = 0.02) was employed to assess the relationship between Hs-CRP and TSHR-Abs levels

In the hyperthyroid state (G1), statistically significant correlation was confirmed between Hs-CRP and Tg-Abs (r = −0.22, *P* = 0.0016), Hs-CRP and TPO-Abs (r = −0.26, *P* < 0.001), and also between Hs-CRP and TSHR-Abs (r = −0.18, *P* = 0.02; Figs 2, 3, 4).

In euthyroid patients (G2), no statistically significant correlation was observed: Hs-CRP and Tg-Abs (*r* = −0.18, *P* = 0.13), Hs-CRP and TPO-Abs (*r* = −0.17, *P* = 0.15), Hs-CRP and TSH (*r* = 0.01, *P* = 0.91), Hs-CRP and TSHR-Abs (*r* = −0.19, *P* = 0.17).

**Fig. 5:**
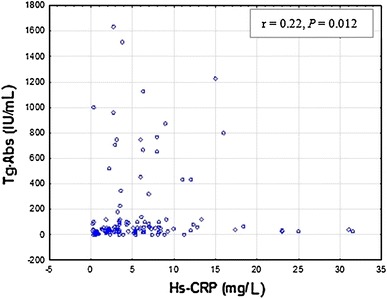
Correlations between serum Hs-CRP and Tg-Abs levels in hypothyroid patients (G3). Tg-Abs 0–34 IU/mL; Hs-CRP <3 mg/L. Spearman’s rank correlation (*r* = 0.22, *P* = 0.012) was employed to assess the relationship between Hs-CRP and TPO-Abs levels

**Fig. 6:**
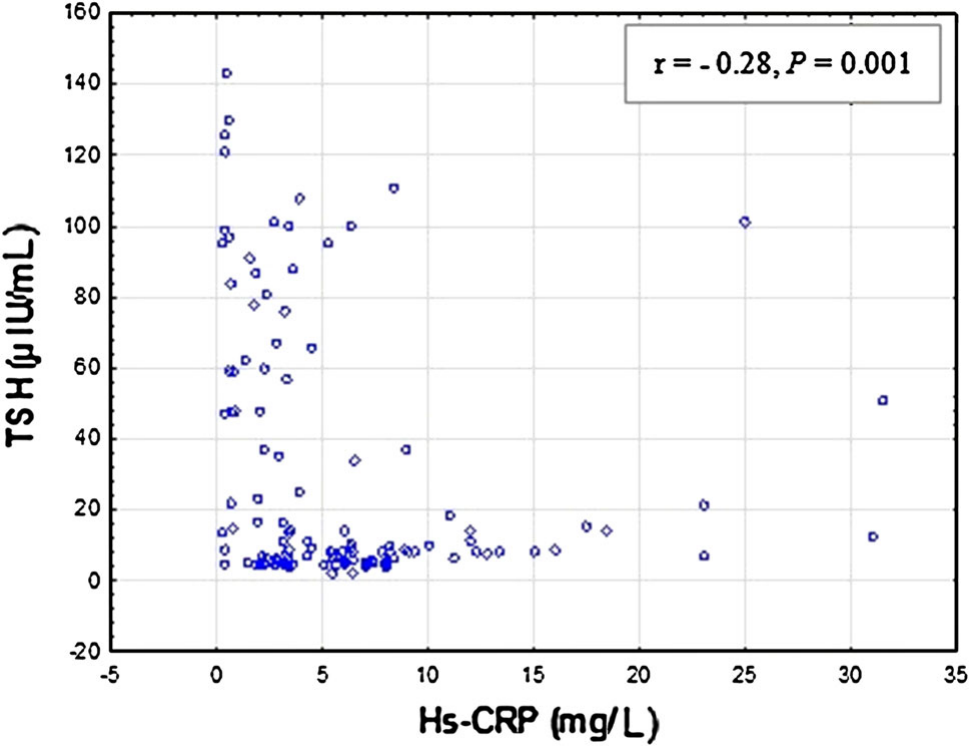
Correlations between serum Hs-CRP and TSH levels in hypothyroid patients (G3). Serum TSH concentration a third-generation sensitivity ≤0.005 IU/mL; Hs-CRP <3 mg/L. Spearman’s rank correlation (*r* = −0.28, *P* = 0.001) was employed to assess the relationship between Hs-CRP and TSH levels

In the hypothyroid state (G3), a statistically significant correlation was confirmed between Hs-CRP and Tg-Abs (*r* = 0.22, *P* = 0.012) and also Hs-CRP and TSH (*r* = −0.28, *P* = 0.001) (Figs. 5, 6). No statistically significant correlation was observed between Hs-CRP and TPO-Abs (*r* = 0.20, *P* = 0.06) as well as Hs-CRP and TSHR-Abs (*r* = −0.23, *P* = 0.11).

## Discussion

Hs-CRP is a valuable tool in determining the prognosis of systemic inflammatory diseases such as rheumatoid arthritis (Graf et al. 2009) and active systemic vasculitis (Tomasson et al. 2011). Limited inflammatory disorders, such as Crohn’s disease, are better managed with the implementation of a regular Hs-CRP measurement (Erlinger et al. 2004; Owczarek et al. 2012). Hs-CRP is also useful in determining renal transplant rejection (Grebe et al. 2011; Roshdy et al. 2012). A moderate increase in the concentration of Hs-CRP occurs in liver diseases (Komoriya et al. 2012), autoimmune diseases (Rezaieyazdi et al. 2011), and neoplasms (Hopkins et al. 2012). Cigarette smoking, obesity, aging (Yu and Rifai 2000) and hormone replacement therapy in postmenopausal woman (Ridker et al. 2000) are all associated with minor increases in Hs-CRP levels. This protein serves a specific function in the risk of developing cardiovascular disease in which inflammation plays a significant contributory role (Järvisalo et al. 2006). Cardiovascular disease is seen more frequently in both hyperthyroidism and hypothyroidism. Hyperthyroidism increases heart rate and cardiac output leading to an increased risk of atrial fibrillation and heart failure, with a threefold increase of coronary events seen in patients with increased triiodothyronine (T3) levels (Peters et al. 2000). The increased risk of cardiovascular disease may also be observed in subclinical hyperthyroidism (Kaminski et al. 2012; Metso et al. 2007; Parle et al. 2001), as well as in patients previously treated with RAI (Franklyn et al. 1998). Hypothyroidism lowers heart rate and cardiac output leading to increased risk of cardiovascular disease (Biondi et al. 2002; Biondi 2012), hypercholesterolemia, and hypertension (Klein and Ojamaa 2001; Toft and Boon 2000). Even subclinical hypothyroidism already presents a doubling of myocardial infarction and an elevated rate of atherosclerosis (Hak et al. 2000). It should be emphasized that patients suffering from thyroid cancer prior to RIT who discontinue l-thyroxine for five weeks are also at risk of cardiovascular disease. Therefore, the best solution would be to use Thyrogen (Thyrogen^®^, Genzyme, USA) therapy in all patients, which is not always possible on account of the costs involved. An analogous situation is observed in cases of chronic hypothyroidism after RIT in benign thyroid disorders. Though this is not always a consequence, patients should nevertheless be informed about the possibility of its occurrence and that it is also associated with the risk of cardiovascular disease.

The mechanism of CRP elevation in hypothyroidism is unknown. Several signs and symptoms in patients with hypothyroidism suggest an abnormality of inflammation. These are thought to be the result of an interaction of IL-6 on TNF-α and IL-1. This interaction results from the elevated CRP in hypothyroidism. In the study, the degree of elevation of CRP in plasma was not correlated with the severity of hypothyroidism. Other underlying mechanisms of CRP increase in both hyper- and hypothyroidism remain unclear. Except for the above-mentioned cytokines, lack of thyroid hormones leads to slowing down the overall metabolic rate, and all the biochemical processes may be impaired under those circumstances. Thus, the rate of CRP clearance may result in CRP serum level increase. Similarly, slow CRP uptake in target cells might also add to this phenomenon. In contrast, hyperthyroidism results in rapid metabolic activity, which may lead to the hyperactivity of adrenergic nervous system, stimulation of immune system and significantly increased peripheral blood flow: all those conditions might result in an increase of CRP concentration.

The present study found a statistically insignificant correlation between TSH elevation and the increased Hs-CRP level in euthyroid patients (G2), as previously observed by Tanda et al. (2008), whereas other studies found CRP not to be elevated in hypothyroidism (Christ-Crain et al. 2003; Jublanc et al. 2004), or in hyperthyroidism (Pearce et al. 2003). The discrepancies between our results and the results cited above may have diverse causes, including sample size, age, and sex.

To the best of our knowledge, this is the first report to assess CRP levels in different forms of thyroid disease, which include toxic adenoma, hyperthyroidism and hypothyroidism subsequent to the use of drugs such as IFN-α and AM, and thyroid cancer patients. Although, Pearce et al. (2003) had examined 353 patients with various thyroid conditions (AIT, subacute thyroiditis, toxic diffuse goiter, nodular goiter, HT, acute hypothyroidism, and postpartum thyroiditis), there are no reported studies on the assessment of Hs-CRP changes in thyroid dysfunction, except for a few that demonstrated elevated CRP in hypothyroidism (Pearce et al. 2003; Tanda et al. 2008). In our study, we observed particularly high concentrations of Hs-CRP in patients with hypothyroidism after RIT (G3: L), with the highest concentrations seen in postpartum thyroiditis (G3: N). It would be of great interest to see the levels of Hs-CRP in other types of thyroiditis, particularly in subacute thyroiditis. Unfortunately, a limitation of this study is that patients with subacute thyroiditis were not included as the number of patients was insufficient to perform statistics (one patient).

Another interesting group of patients we examined were the subjects treated for viral hepatitis in whom IFN-α was used. Here, both hyperthyroid and hypothyroid IFN-α-treated patients had Hs-CRP values not different from those detected in the euthyroid group. This finding may lead to some hypothesis that IFN-α treatment might mitigate the impact of thyroid dysfunction on inflammatory processes as measured with Hs-CRP. This hypothesis, however, requires further research to be fully elucidated.

Furthermore, as previously attempted by Pearce et al. (2003), we aimed to use CRP as a diagnostic tool that would help distinguish type I from type II of inflammatory AIT. Interestingly, CRP level was elevated in most cases of both AIT I and II but there was no statistical difference between two types. Therefore, on the basis of our results, we conclude that CRP cannot be used for differentiation between two classes of AIT.

In conclusion, as shown in this paper, the serum CRP concentration is increased in various types of hyperthyroidism and hypothyroidism. This is particularly relevant in postpartum thyroiditis and in patients after RIT. The impact of this situation on human health requires further research, however, one might assume that some types of thyroid disease may lead to systemic inflammatory reactions that are reflected in elevated CRP levels.

## Abbreviations

AIT: Amiodarone-induced thyrotoxicosis
AM: Amiodarone
CRP: C-reactive protein
GD: Graves’ disease
fT4: Free tetraiodothyronine
fT3: Free triiodothyronine
HT: Hashimoto’s thyroiditis
Hs-CRP: High-sensitive CRP
IFN: Interferon
IIH: IFN-α-induced hyperthyroidism
IIT: IFN-α-induced thyrotoxicosis
RIT: Radioiodine therapy
RAIU: Radioactive iodine uptake
TH: Thyroid hormones
TNG: Toxic nodular goiter
TSH: Thyrotropin
TSHR-Abs: Autoantibodies against the thyrotropin receptor
Tg-Abs: Thyroglobulin autoantibodies
TPO-Abs: Thyroperoxidase autoantibodies
